# One‐stage versus two‐stage technique using two splinted extra‐short implants: A multicentric split‐mouth study with a one‐year follow‐up

**DOI:** 10.1111/cid.13113

**Published:** 2022-06-14

**Authors:** Maria Menini, Paolo Pesce, Francesca Delucchi, Giulia Ambrogio, Camilla Canepa, Massimo Carossa, Francesco Pera

**Affiliations:** ^1^ Division of Prosthodontics and Implant Prosthodontics, Department of Surgical Sciences and Integrated Diagnostics (DISC) University of Genoa Genoa Italy; ^2^ Department of Surgical Sciences and Integrated Diagnostics (DISC) University of Genoa Genoa Italy; ^3^ CIR Dental School Department of Surgical Sciences University of Turin Torino Italy

**Keywords:** bone resorption, dental implants, implant stability, short implants

## Abstract

**Objective:**

To compare the clinical outcomes of extra‐short implants (≤6.5 mm) inserted with one‐stage versus two‐stage technique in adjacent sites of the upper or lower jaw.

**Materials and Methods:**

In this split‐mouth multicenter study, implants were randomly divided into two groups according to the healing phase: two‐stage and one‐stage technique. Primary outcome measures were implant survival, implant success, and prosthodontic complications. Secondary outcome measurements were: implant stability quotient (ISQ) collected at surgery time (T0), and after 3 (T3) and 12 (T12) months, marginal bone level (MBL) evaluated at T0, T3, T6, and T12, marginal bone loss evaluated at T6 and T12, plaque index (PI), probing depth (PD), bleeding on probing (BoP) evaluated at T3, T6, and T12. Significances of differences between groups were tested by linear mixed model with random intercept.

**Results:**

Nineteen patients **(**8 males and 11 females) were included. A total of 38 implants were inserted. At T12 implant cumulative survival and implant success rate were 100% in both groups. No statistically significant differences were recorded for any of the analyzed parameters between the two groups at any time point. ISQ values were similar at T0 (two‐stage: mean 67.53 ± SD 19.47; one‐stage: mean 66.53 ± 19.07 *p* = 0.8738) and increased in both groups at the 12‐month follow‐up appointment (two‐stage: 81.1 ± 7.04; one‐stage: 81.39 ± 0.9266). MBL values were similar in the two groups at any time point. At T12 marginal bone loss was 0.46 ± 0.41 (two‐stage) and 0.45 ± 0.38 (one‐stage) mm (*p* = 0.9417), while mean PD was 2.7 ± 0.85 (two‐stage) and 2.69 ± 0.89 (one‐stage) mm.

**Conclusions:**

Within the limits of the present short‐term report, extra‐short implants demonstrated optimal clinical outcomes using the one‐stage technique, without statistically significant differences compared with the traditional two‐stage approach.

**Summary Box**

**What is known on the topic**

Extra‐short implants have been proposed for the rehabilitation of atrophic jaws.

- They provide a less invasive clinical procedure compared to alternative techniques.
- No information is available on clinical outcomes of one‐stage versus two‐stage surgical techniques when using short and extra‐short implants.

**What this study adds**

The one‐stage and the two‐stage surgical approach showed similar outcomes when using splinted extra‐short implants with a one‐year follow‐up.

- A submerged healing of extra‐short implants is not necessary.

## 1 INTRODUCTION

Maxillary/mandibular posterior bone atrophy is a common clinical issue, involving the lack of sufficient residual bone volume for the insertion of dental implants. Several surgical techniques have been developed over the years to face this clinical situation, including guided bone regeneration (GBR), block grafts, sinus lift, bone distractions, alveolar nerve transposition, mediodistally tilted implants or the use of pterygoid or zygomatic implants.^1^, ^2^, ^3^, ^4^, ^5^, ^6^, ^7^ However, these surgical procedures can result in a long treatment time, high costs, and morbidity.^8^, ^9^

For these reasons, short and extra‐short implants have been proposed as a simplified minimally invasive alternative, to adapt implants macrostructure to the existing anatomy, reducing biological and economical costs, treatment time, and increasing patient acceptance.^10^, ^11^

In the Literature there is not a clear definition of “short” implants with cut‐off values. The European Association of Dental Implantologists (BDIZ EDI), at the end of the 11th European Consensus Conference (EuCC) stated that implants can be referred to as “short” if their designed intrabony length measures ≤8 mm with diameters ≥3.75 mm. Standard implants are considered those with lengths >8 mm and diameters of 3.75–4.0 mm. Finally, “ultra‐short” implants are considered to be those with lengths less than 6, 6.5 mm.^12^, ^13^

In the past, the use of short implants has been discouraged from the biomechanical point of view, due to the unfavorable crown‐to‐implant ratio (C/I ratio). However, according to recent literature, C/I ratio seems not directly correlated with peri‐implant bone loss^14^ or biological complications and implant failure,^15^ but it can be related to some prosthetic complications, such as crown or abutment loosening (C/I ratio ≥1.46), or abutment fractures in the posterior jaw (C/I ratio ≥2.01).^15^

To increase bone to implant contact (BIC) when using short implants, it has been suggested to increase the implant diameter, with consequent better withstanding of occlusal stresses and loads distribution on peri‐implant bone. This has been suggested as an important factor to guarantee successful long‐term results.^16^

Another aspect to be explored in the case of short and extra‐short implants is the surgical protocol. In implant dentistry, two procedures can alternatively be adopted: one‐stage or two‐stage technique.^17^ According to the two‐stage approach, once the implant has been inserted, the mucosal flaps are sutured above it and a submerged healing occurs avoiding the risk of micromovements of the implant during osseointegration. Once the implant is osseointegrated, a second surgery is needed to uncover the implant and proceed with the prosthodontic rehabilitation. The one‐stage approach consists of screwing an implant healing abutment (of variable height, based on the thickness of the mucosa) on the implant at the time of surgery. This abutment immediately establishes a connection with the oral cavity, and provides the nonsubmerged healing of the implant, avoiding the need of a second‐time surgery for the prosthodontic phase.

According to recent Literature, when a sufficient primary stability is present, there is no difference in the outcomes between the two approaches as regard peri‐implant bone loss.^18^ However, no information is available in the literature regarding submerged versus transmucosal healing of short and extra‐short implants.

The aim of the present multicenter prospective clinical trial is to compare the clinical outcomes of extra‐short implants inserted with one‐stage versus two‐stage technique in the upper or lower jaw in adjacent sites. The null hypothesis tested was that no differences exist in survival rate, bone resorption, implant stability and periodontal indexes using extra‐short implants with a one‐stage or a two‐stage surgical approach.

## 2 MATERIALS AND METHODS

### 2.1 Patients' selection and study design

The present research was designed as a split‐mouth randomized controlled trial. From November 2019 to January 2020, 10 consecutive patients referred to the Division of Prosthodontics and Implant Prosthodontics (Department of Surgical Sciences – DISC) of the University of Genoa and 11 referred to the Prosthodontics Department of the Dental School of the University of Turin, Italy, were recruited. All the selected patients required the insertion of extra‐short implants and they satisfied the inclusion/exclusion criteria reported below. The present research was performed following the Declaration of Helsinki and was approved by the local ethical committee (CER Liguria, ref. number 254/2019 – DB id 4648). All the participants signed an informed consent, and the study was conducted in compliance with the CONSORT EQUATOR guidelines.

### 2.2 Inclusion/exclusion criteria

The inclusion criteria were:

- Age ≥ 18 years old.
- Two adjacent missing teeth in the posterior maxilla or mandible with a reduced bone quantity (vertical distance between bone crest to the maxillary sinus or inferior alveolar nerve ≤8 mm).
- Adequate bone availability to insert 2 implants with a 5.0‐ or 5.5‐mm diameter, and 5.5–6.5 mm long.
- Full‐mouth plaque Index (FMPI) and Full‐mouth bleeding index (FMBI) < 10% assessed in six points for each tooth using a periodontal UNC 15 probe (Hu‐Friedy, Chicago, IL, USA).
- Patients must be willing to participate and to attend the planned follow‐up visits

Exclusion criteria were:

- Medical condition contraindicating implant surgery.
- Smoker of more than 10 cigarettes per day, cigar equivalents. or tobacco chewers.
- Local inflammation (included untreated periodontitis).
- Post‐extraction sites with less than 6 months of healing.
- Bruxism.

### 2.3 Implant surgery

One hour before implant surgery patients were instructed to take antibiotic (Amoxicillin 2 g – Amoxicillina EG – Biopharma S.r.l. – Roma, Italy). Chlorhexidine 0.2% (Curasept S.p.A., Saronno, Italy) was used to rinse the mouth for 1 minute immediately before surgery. All patients were treated by the same calibrated and experienced dentists (FP, PP, and IC) specialized in implant surgical treatment. After local anesthesia (4% articaine with 1:100 000 adrenaline; Alfacaina SP; Dentsply Italy, Rome, Italy), a crestal incision was performed, and a full‐thickness flap elevated. The osteotomy was conducted following the manufacturer instruction, using low‐speed drilling without irrigation following the drilling procedure described by Anitua and colleagues.^19^ Two adjacent implants were inserted in each patient (Interna, BTI Biotechnology Institute, Vitoria, Spain). All the implants had a 5.0 or 5.5 mm diameter and were 5.5 or 6.5 mm long.

The implant platform was positioned at the bone level. Insertion torque and implant stability quotient (ISQ) were collected (Osstell, W&H Co., Bürmoos, Austria Company).

A pre‐generated random sequence was created (Random number generation pro 1.91 for Windows, Segobit software; Segobit, Moscow, Russia, http://www.segobit.com) by one operator (PP). Opaque sealed envelopes were prepared, and one implant of each patient was randomly assigned to the one‐stage group. Envelopes were opened at this time to randomly choose the one‐stage implant and the two‐stage one: on the one‐stage implant, a cover screw was inserted to allow a submerged healing (one‐stage approach) while on the two‐stage implant, a straight Multi‐Im® abutment (BTI Biotechnology Institute, Vitoria, Spain) and a healing abutment were screwed on the implant to create a transmucosal healing. Flaps were therefore repositioned and sutured to obtain optimal adaptation of the mucosa to the titanium abutment.

**FIGURE 1:**
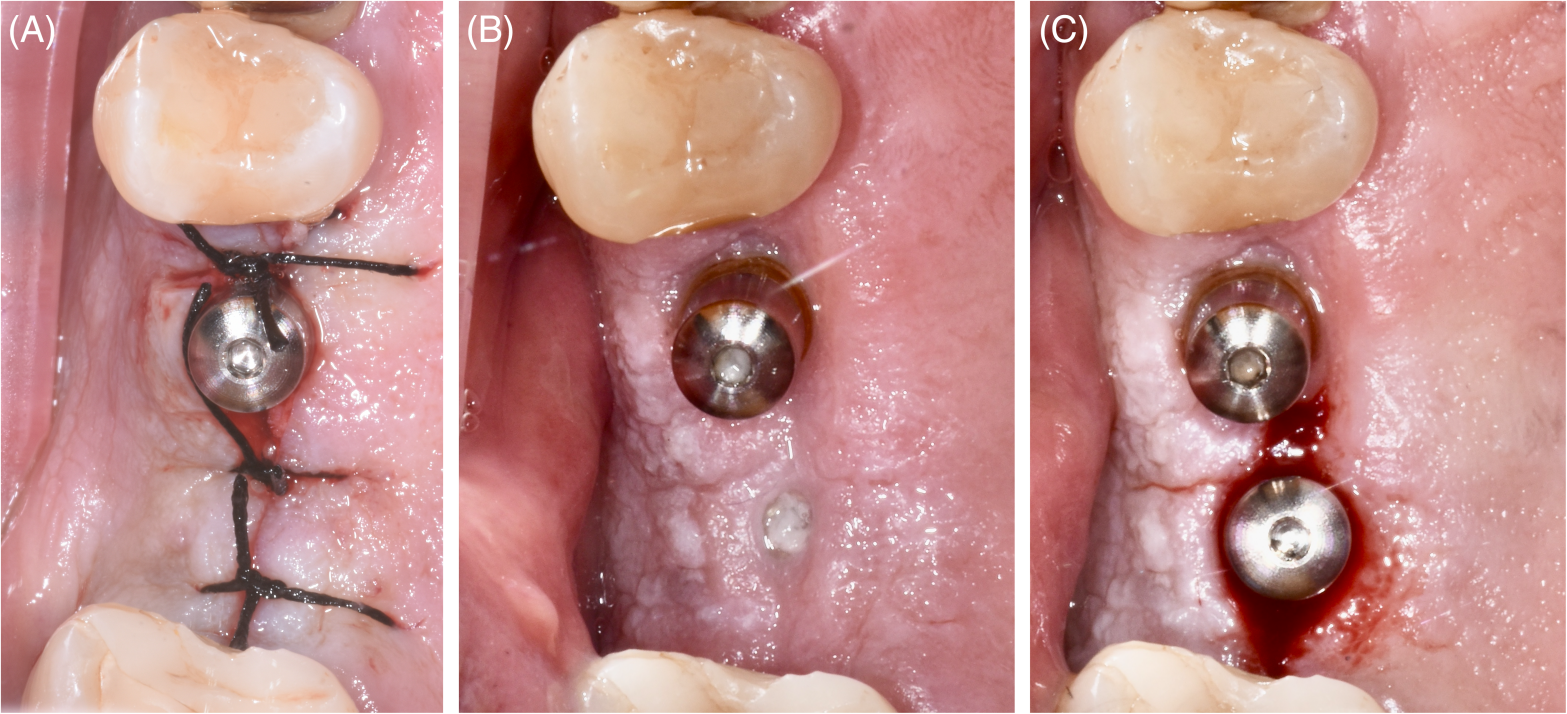
Intraoral images of one of the patients included in the present research: at T0 immediately after implant insertion (on the left); at the sutures removal (in the middle) and at T3, at the second surgery of the submerged implant (on the right)

After 3 months, a second surgery was performed to connect the Multi‐Im® abutment to the two‐stage implant. Two weeks later, impressions for the definitive prostheses were made using the pick‐up impression technique with polyether impression material (3M™ Impregum™, St. Paul, MN). Two screw‐retained splinted crowns endowed with a metal framework and a composite resin veneering material were screwed on the Multi‐Im® abutments (Figure 1).

The supportive implant maintenance program provided a follow‐up appointment at least every 6 months.

### 2.4 Outcome measures

The primary outcome measures were implant survival (cumulative survival rate – CSR), implant success and prosthodontic complications (i.e., chipping, fracture, screw loosening).

In the present study, an implant was considered survived if it was in place and it has not been lost. A survived implant was considered successful if it was immobile when tested individually; it did not preclude the placement of the planned functional and esthetic prosthesis that was satisfactory to both patient and clinician; there was no pain, discomfort, altered sensation, or infection attributable to the implant; and the mean vertical bone loss was less than 1.5 mm during the first year and 0.2 mm annually after the first year of function.

**FIGURE 2:**
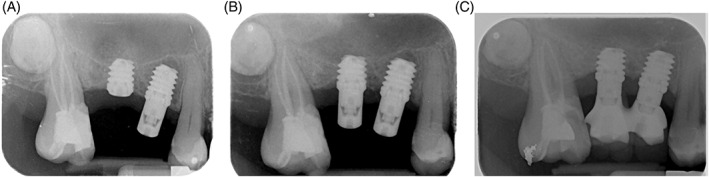
Intraoral radiographs of one of the patients included in the present research: at T0 immediately after implant insertion (on the left); at T3 (in the middle) and at T12, 1 year after implant insertion (on the right)

Secondary outcome measures were:

- ISQ value: collected at T0 (immediately after implant insertion), and after 3 (T3) and 12 (T12) months.
- Marginal Bone level: evaluated at T0, T3, T6, and T12 using intraoral digital periapical radiographs taken with the parallel long‐cone technique (Figure 2). Measurements were done using the implant–abutment interface as a reference point. Interproximal bone levels were assessed from these reference points to the most coronal bone levels at the mesial and distal side of each implant using a digital software (OrisWin DG, FONA, Assago, Italy).
- Marginal bone loss was measured as the difference between bone level at T6 and T12 and bone level at the time of implant insertion (T0).
- Periodontal indexes: (PI, PD, and BoP) were assessed in four points for each implant using a periodontal UNC 15 probe (Hu‐Friedy, Chicago, IL, USA). BoP was evaluated as the presence of bleeding (yes/no); PI was defined as the presence of plaque (yes/no) on the Multi‐Im® abutment or prosthesis using an erythrosine gel. These were evaluated at T3 for test implants only and at T6 and T12 for all implants.

One author from each Center (F.D. and G.A.) performed all the clinical measurements. The examiners were trained and calibrated before the start of the clinical evaluation. Cohen's kappa statistic was used to calculate observer agreement. Excellent intra‐observer (kappa values of 0.78 and 0.80) and interobserver (a kappa value of 0.80) agreement was recorded in this study.

### 2.5 Statistical analysis

Means with standard deviations were reported for the quantitative parameters recorded following normal distribution. For parameters not following a normal distribution median, maximum and minimum were reported. Longitudinal assessment of ISQ, marginal bone loss, PD, BoP, and PI during follow‐up was performed using a linear mixed model with random intercept after visual inspection of their probability distribution. In all these regression models, the dependent variable was the outcome and the independent variables were the time indexes, the treatment group and their interaction. Additionally, an analysis comparing maxilla data versus mandible data was conducted. A significance level of 5% was adopted in all tests and SPSS IBM software (version 25) was used.

A post hoc analysis was conducted to evaluate the power of our data on the bone resorption at T12.

## 3 RESULTS

Twenty‐one patients were screened for inclusion in the present research. Two of them were excluded for medical conditions contraindicating implant surgery. Finally, 19 patients meeting the inclusion/exclusion criteria were enrolled in the study: 9 at the University of Genoa and 10 at the University of Turin; 38 implants were inserted (18 in the mandible, 20 in the maxilla). Eight patients were males and 11 females; the mean age was 62 (11) years (range: 38–82 years). Two patients were smokers. There were no drop‐outs throughout the study period and all the patients anecdotally reported to be satisfied with their implant rehabilitation. At the end of the study, 38 implants were examined. Implant CSR was 100% in both groups and no surgical, post‐surgical, or prosthodontic complication occurred. All the implants were considered successful.

The main periodontal parameters are reported in Table 1. Table 2 reports parameters not following a normal distribution. No statistically significant differences were recorded between the two groups for any of the analyzed parameters at any time point. ISQ values were similar at T0 (two‐stage: 67.53 (19.47); one‐stage: 66.53 (19.07) *p* = 0.87) and increased in both groups at the 12‐month follow‐up appointment (two‐stage: 81.1 (7.04); one‐stage: 81.39 (6.07) *p* = 0.93). No statistically significant differences were identified in marginal bone loss at any time point. At 6 months, mean bone loss was 0.37 (0.38) mm in the one‐stage and 0.41 (0.28) mm in the two‐stage group (*p* = 0.69). At the 12‐month follow‐up visit, bone loss was 0.46 (0.41) mm in the two‐stage group and 0.45 (0.38) mm in the one‐stage group (*p* = 0.94). Also, PI, BoP, and PD were similar between the two groups at any time point.

**TABLE 1:** Main outcomes

Parameter	Two‐stage mean (*SD*)	One‐stage mean (*SD*)	Statistical significance
ISQ T0	67.53 (19.47)	66.53 (19.07)	0.87
ISQ T3	78.26 (8.76)	79.26 (7.88)	0.71
ISQ T12	81.1 (7.04)	81.39 (6.07)	0.93
Insertion torque (Ncm)	37 (16)	43 (14)	
BoP T3	—	0.53 (0.9)	—
BoP T6	0.47 (0.7)	0.42 (0.69)	0.83
BoP T12	1.06 (1.34)	0.71 (1.10)	0.41
Marginal bone level T0 (mm)	0.09 (0.21)	0.12 (0.23)	0.68
Marginal bone level T3 (mm)	0.35 (0.34)	0.37 (0.35)	0.86
Marginal bone level T6 (mm)	0.45 (0.34)	0.53 (0.32)	0.48
Bone resorption T6‐T0 (mm)	0.37 (0.38)	0.41 (0.28)	0.69
Marginal bone level T12 (mm)	0.61 (0.34)	0.65 (0.38)	0.21
Bone Resorption T12‐T0 (mm)	0.46 (0.41)	0.45 (0.38)	0.94
PI T3	—	0.42 (0.96)	—
PI T6	0.89 (1.10)	0.79 (1.08)	0.78
PI T12	1.06 (1.14)	0.94 (1.09)	0.76
PD T3 (mm)	—	2.37 0.96)	—
PD T6 (mm)	2.45 (0.85)	2.34 (1.00)	0.11
PD T12 (mm)	2.7 (0.85)	2.69 (0.89)	0.97

Abbreviations: BoP, bleeding on probing; ISQ, implant stability quotient; PD. probing depth; PI, plaque index; SD, standard deviation.

**TABLE 2:** Descriptive statistics of variables that did not follow a normal distribution

		ISQ T0	BoP T3	BoP T6	BoP T12	Marginal bone level T0	Marginal bone level T3	PI T3	PI T6	PI T12
C	Median	73.50	0.00	0.00	0.00	0.00	0.42	0.00	1.00	1.00
	Minimum	20.00	0.00	0.00	0.00	0.00	0.00	0.00	0.00	0.00
	Maximum	87.50	3.00	2.00	4.00	0.665	0.985	4.00	3.00	4.00
T	Median	77.00	0.00	0.00	0.00	0.00	0.30	0.00	0.0000	0.00
	Minimum	20.00	0.00	0.00	0.00	0.00	0.00	0.00	0.00	0.00
	Maximum	85.50	3.00	2.00	4.00	0.795	1.025	4.00	3.00	3.00
Statistical significance		0.92	0.93	0.77	0.49	0.34	0.95	0.25	0.73	0.80

Differences among maxilla and mandible are reported in Tables 3 and 4. Statistically significant differences were present among ISQ T0 (*p* = 0.004), ISQ T3 (*p* = 0.07), insertion torque (*p* = 0.00) and BoP T12 (*p* = 0.03) with higher values in the mandible than in the maxilla. No differences were recorded for any other parameter.

**TABLE 3:** Maxilla versus Mandible. Descriptive statistics of variables that did not follow a normal distribution

		ISQ T0	ISQ T12	BoP T3	BoP T6	Marginal bone level T0	Marginal bone level T3	Marginal bone level T12	PI T3	PI T6
mandible	N	18	10	18	18	18	18	16	18	18
	Median	78.00	85.25	0.00	0.50	0.00	0.33	0.70	0.00	1.00
	Minimum	21.00	69.00	0.00	0.00	0.00	0.00	0.00	0.00	0.00
	Maximum	87.50	87.50	2.00	2.00	0.80	1.03	1.46	4.00	3.00
maxilla	N	20	8	20	20	20	20	18	20	20
	Median	69.75	82.25	0.00	0.00	0.00	0.34	0.62	0.00	0.00
	Mínimum	20.00	68.50	0.00	0.00	0.00	0.00	0.00	0.00	0.00
	Maximun	81.00	85.00	3.00	2.00	0.67	1.03	1.13	3.00	3.00
Statistical significance		0.004	0.07	0.47	0.05	0.72	0.89	0.28	0.50	0.33

**TABLE 4:** Maxilla versus Mandible. Descriptive statistics of variables follow a normal distribution

		N	Mean	SD	Statistical significance
ISQ T3	mandible	18	82.31	5.46	0.01
	maxilla	20	75.58	9.09	
Insertion torque (Ncm)	mandible	18	50.28	12.18	0.00
	maxilla	20	30.75	11.50	
BoP T12	mandible	16	1.38	1.26	0.03
	maxilla	18	0.44	1.04	
Marginal bone level T6	mandible	18	0.55	0.34	0.29
	maxilla	20	0.44	0.31	
Bone resorption T6‐T0	mandible	18	0.47	0.35	0.16
	maxilla	20	0.32	0.30	
Bone resorption T12_T0	mandible	18	0.54	0.45	0.21
	maxilla	20	0.38	0.33	
PI T12	mandible	16	0.88	0.96	0.54
	maxilla	18	1.11	1.23	
PD T3	mandible	18	2.53	1.10	0.75
	maxilla	20	2.43	0.71	
PD T6	mandible	18	2.41	0.98	0.94
	maxilla	20	2.38	0.89	
PDT 12	mandible	16	2.78	0.78	0.57
	maxilla	18	2.61	0.93	

The post hoc analysis conducted on bone resorption revealed a power of 96%. The difference of means is equal to the Equivalence limit by a two‐sided T‐Student Test (of Equivalence) for two independent samples, taking into account that the significance level is 5.00%, and assuming that the Equivalence limit is 0.50, the mean of the Reference group is 0.45 mm, the mean of the Experimental group is 0.46 mm, and the standard deviation of both groups is 0.40 mm.

## 4 DISCUSSION

The aim of the present research was to compare the clinical outcomes of extra‐short implants inserted with one‐stage versus two‐stage technique in adjacent sites of the upper or lower jaw. To the authors' knowledge, this is the first split‐mouth clinical study addressing this topic. At the 1‐year follow‐up appointment, all the implants were osseointegrated and successfully in function in both groups and no complications occurred. Mean bone loss was within normal limits and must be probably accounted for initial physiological bone remodeling.

Based on the results of this work, the null hypothesis can be rejected since no significant differences were identified between one‐stage and two‐stage groups for any of the analyzed parameters.

In the last years, different techniques have been proposed to rehabilitate in a fast and predictable way patients with low availability of native residual bone avoiding invasive surgical augmentation procedures.^13^, ^20^, ^21^ Among these, tilted implants and short implants represent the most used approaches. Mesiodistally tilted implants have been introduced in the clinical practice, to use all the available native bone and to preserve anatomical structures, such as the maxillary sinus and the mandibular nerve.^22^, ^23^ This is an option especially proposed in full‐arch immediate loading rehabilitations, when a reduced amount of bone is present in distal areas and longer implants are preferable to increase primary stability.^22^ However, different biomechanical conditions must be taken into account in partial and delayed loading rehabilitations.^24^ A finite element (FEM) study by Bevilacqua and colleagues^25^ showed that when using single implants, the higher is the implant inclination, the higher is the stress transferred to peri‐implant bone. Additionally, according to a FEM simulation by Anitua and colleagues^16^ increasing the implant diameter, it is possible to reduce the maximum von Mises stress in peri‐implant bone in the range of 20–30%. This study also showed that occlusal forces are mainly concentrated at the first three threads of the implant, and significantly decrease beyond this level. However, as implant diameter increases (in implants with a 5 or 5.5 mm diameter), the stress around the implant neck decreases, being better dissipated along the bone‐implant interface. As a consequence, the effect of implant diameter on stress distribution in bone was considered more significant than the effect of the implant's length or its geometry by Anitua and colleagues.

One of the most criticized aspects of the use of short implants has been the unfavorable crown‐to‐implant ratio (C/I). In a natural tooth, while the bone is progressively reabsorbed, the fulcrum moves apically, and the tooth becomes more susceptible to harmful lateral occlusal forces. However, according to Misch,^26^ in the case of osseointegrated dental implants, the length is not directly related to the capacity of withstanding lateral forces, and should not be considered a predictor of implant survival, because the implant is ankylosed to bone and does not have any center of rotation two‐thirds down the endosteal/root portion. As previously reported, the greatest part of occlusal forces is concentrated in the first coronal threads of osseointegrated implants.^16^

A systematic review by Meijer and colleagues^14^ concluded that a C/I ranging from 0.86 to 2.14 of single‐tooth, nonsplinted, implants did not demonstrate a high occurrence of biological or technical complications. The authors of this review underlined that the distinction between splinted and nonsplinted crowns can have possible effects on the C/I ratios assessment.

These findings have been confirmed at the “V Consensus Conference of the European Association for Osseointegration – EAO”,^27^ which concluded that a crown of double the length of the implant fixture is not associated with biological complications in splinted or nonsplinted crowns. However, it is the authors opinion that when a multiple‐implant restoration is planned, splinting with a rigid framework is the best option to favor a more even stress distribution among supporting implants and this should be even more so when using extra‐short implants.^24^, ^28^, ^29^, ^30^ According to this concept, all the rehabilitations in the present study were splinted crowns.

Taking into consideration, the surgical/prosthodontic technique employed, a recent systematic review stated that implants placed with a nonsubmerged technique have a feasible higher risk (2%) of early failure, but the power of the evidence about the effects on bone loss was low, favoring nonsubmerged healing.^18^

In contrast with the above‐mentioned study and in agreement with our outcomes, a randomized study on 140 patients and 310 implants found that there was no statistically significant difference between the two techniques as regards bone loss.^31^

When a submerged healing is guaranteed following the two‐stage approach, implant micromotion that might jeopardize osseointegration is avoided. On the other side, when a transmucosal component (such as the Multi‐Im® abutment in the present study) is screwed on the implant immediately after surgery, an hermetic matching of two prefabricated components (the implant and the transmucosal abutment) is realized. Peri‐implant soft tissue will heal in direct contact with the abutment and the mucosal seal will not be disrupted during subsequent prosthodontic phases.

Gentile and colleagues found higher success rates for short implants (6 mm wide × 5.7 mm) with the two‐stage rather than the one‐stage technique.^32^ However, a recent retrospective study by Kim and colleagues on short implants (<8 mm) found no differences between these two surgical approaches in terms of mean bone loss, implant survival and success rate.^33^ Anitua and colleagues^34^ in a retrospective study with a 15‐year follow‐up found that the surgical approach (1‐stage vs 2‐stage) did not significantly affect implant survival (15‐year CSR: 90.2%) and marginal bone loss of short implants in full‐arch rehabilitations. However, the outcomes might not be the same when dealing with partial instead of complete dentures and when using extra‐short instead of short implants, due to different biomechanical implications.

It is known from classical studies such as the one by Fiorellini and colleagues that with nonsubmerged implants, the greatest amount of bone loss occurs immediately after implant placement and then a consistent level is obtained following the period for osseointegration (from week 12).^35^ On the other hand, submerged implants loose peri‐implant bone at different time intervals when compared with the nonsubmerged group. Initially, following implant insertion and within the osseointegration period (weeks 0–12), there is minimal bone loss on these implants. Following the reentry surgery and attachment of transmucosal abutment, a bone resorption occurs due to the formation of the supracrestal tissue height. The present study did not find any difference in bone resorption between extra‐short implants inserted with a one‐stage versus a two‐stage technique at any time point. A possible explanation is the small sample size which made small changes in bone loss insignificant and individual characteristics such as biotype and periodontal status.^36^, ^37^ It must be noted that bone resorption seems to be reduced around short implants.^12^, ^38^ Additionally, it must be noted that a mini‐invasive bone drilling technique has been used according to the implant producers' instruction and this might have minimized the initial bone remodeling subsequent to the surgical trauma.

The possibility to apply a one‐stage technique also when using extra‐short implants brings several advantages, including less morbidity, more patient's comfort, reduced chairside time, and reduced costs. However, these outcomes must be taken with caution since the sample size of the present study was small due to the difficulties in finding patients meeting the strict inclusion/exclusion criteria and several confounding factors might have affected the clinical outcomes (i.e., peri‐implant phenotype conditions, such as soft tissue thickness, amount of keratinized tissue, vertical tissue height, remaining amount of bone surrounding the implants after placement;implant length, that is 5.5 or 6.5 mm, implant diameter, implant distance, types of occlusion, parafunctions, crown/implant ratio, etc.). In addition, x‐rays acquisition was not standardized using a customized support, and due to the study protocol, neither the patients nor the operators were blind to control/test group. Only short‐term outcomes (1 year) have been reported. However, it should be expected that subsequent changes in clinical outcomes will not be related to the surgical technique. Additionally, a sample size calculation was not done before the beginning of the study, however, a post hoc analysis was performed. In favor of the present study, it must be emphasized that a rigorous split‐mouth design has been adopted, with identical implants placed in both sites, therefore, the only difference between one‐stage and two‐stage group was the type of healing (transmucosal vs submerged). This study design was aimed at reducing possible bias and the effect of confounding factors.

## 5 CONCLUSIONS

Based on the 1‐year outcomes of the present investigation, the one‐stage surgical approach can be successfully applied when using two splinted extra‐short implants without significant differences in clinical outcomes compared with the two‐stage approach.

## AUTHOR CONTRIBUTIONS

**Maria Menini**: concept/design, acquisition, analysis of data, drafting the paper, and final approval. **Paolo Pesce**: concept/design, analysis of data, drafting the paper, and final approval. **Francesca Delucchi**: acquisition, drafting the paper and final approval. **Giulia Ambrogio**: analysis of data, statistical analysis, drafting the paper, and final approval. **Massimo Carossa**: concept/design, analysis of data, statistical analysis, drafting the paper, and final approval. **Camilla Canepa**: concept/design, drafting the paper, and final approval. **Francesco Pera**: acquisition, analysis of data, drafting the paper and final approval. All the authors gave a substantial contribution to the conception or design of the work; or the acquisition, analysis, or interpretation of data for the work; AND^2^ drafting the paper or revising it critically; AND^3^ final approval of the version to be published; AND^4^ agreement to be accountable for all aspects of the work in ensuring that questions related to the accuracy or integrity of any parts of the work are appropriately investigated and resolved.

## CONFLICT OF INTEREST

BTI Biotechnology Institute (Spain) provided part of the materials and equipment necessary for the conduction of the present research.

All the authors declare no conflict of interest.

## Data Availability

Data available on request
